# The Effects of Early-Life Stress on Liver Transcriptomics and the Protective Role of EPA in a Mouse Model of Early-Life-Stress-Induced Adolescent Depression

**DOI:** 10.3390/ijms241713131

**Published:** 2023-08-23

**Authors:** Jinlan Zhao, Lihong Ye, Zuyi Liu, Jiayi Wu, Di Deng, Lin An, Shasha Bai, Lei Yang, Binjie Liu, Yafei Shi, Zhongqiu Liu, Rong Zhang

**Affiliations:** 1 Guangdong Provincial Key Laboratory of Translational Cancer Research of Chinese Medicines, Joint International Research Laboratory of Translational Cancer Research of Chinese Medicines, International Institute for Translational Chinese Medicine, School of Pharmaceutical Sciences, Guangzhou University of Chinese Medicine, Guangzhou 510006, China; zhaojinlan@gzucm.edu.cn (J.Z.); yelihongyyy@163.com (L.Y.); liuzuyi1999@163.com (Z.L.); dengdi@gzucm.edu.cn (D.D.); anlin@gzucm.edu.cn (L.A.); baishasha@gzucm.edu.cn (S.B.); yanglei@gzucm.edu.cn (L.Y.); 20201110556@stu.gzucm.edu.cn (B.L.); 2 School of Fundamental Medical Science, Guangzhou University of Chinese Medicine, Guangzhou 510006, China; wujiayi19980809@163.com (J.W.); shiyafei@gzucm.edu.cn (Y.S.)

**Keywords:** early-life stress, adolescent depression, eicosapentaenoic acid, liver

## Abstract

Early-life stress (ELS) was found to increase the risk of adolescent depression, and clinical evidence indicated that eicosapentaenoic acid (EPA) was decreased in patients with adolescent depression, but the underlying mechanisms are unclear. Here, we utilized an ELS model of maternal separation with early weaning to explore the protective role of EPA in adolescent depression. We found that that ELS induced depression-like behavior rather than anxiety-like behavior in adolescent mice. RNA-sequencing results showed that ELS changed the transcription pattern in the liver, including 863 upregulated genes and 971 downregulated genes, especially those related to the biosynthesis of unsaturated fatty acids metabolism in the liver. Moreover, ELS decreased the expression of the rate-limiting enzymes, fatty acid desaturases 1/2 (FADS1/2), involved in the biosynthesis of EPA in the liver. Additionally, ELS reduced the levels of EPA in the liver, serum, and hippocampus, and EPA administration improved depression-like behavior-induced by ELS. Our results provide transcriptomic evidence that ELS increases the risk of adolescent depression by reducing the synthesis of unsaturated fatty acids in the liver, especially EPA, and suggest that supplementation with EPA should be investigated as a potential treatment for adolescent depression.

## 1. Introduction

Early-life stress (ELS), such as maternal separation, childhood social failure, abuse, and neglect, has been found to be strongly associated with an increased risk of depression in adolescence [1]. Notably, in China, 26.6% of children and adolescents under 18 years of age have experienced physical abuse, 19.6% have experienced emotional abuse, 8.7% have experienced sexual abuse, and 26.0% have been neglected [2]. However, the mechanisms underlying the induction of depressive damage by ELS remain unclear. Furthermore, the antidepressants currently employed in clinical treatment regimens often come with a host of side effects, such as nausea, vomiting, discontinuation due to adverse events, extrapyramidal side effects, and weight gain [3,4,5]. For instance, it has been reported that various antidepressants, such as duloxetine, nefazodone, paroxetine, sertraline, and vilazodone, are associated with increased incidence of nausea and vomiting [5]. In addition, while fluoxetine and aspirin have received approval for treating adolescent depression, their usage has been linked to a heightened risk of suicide-related adverse events [4,6]. Therefore, investigating the mechanisms underlying ELS-induced adolescent depression and identifying effective drugs to treat adolescent depression have significant social implications.

Recent studies have shown that the pathogenesis of adolescent depression is primarily related to disturbances in the metabolism of polyunsaturated fatty acids (PUFAs) and purines, while the pathogenesis of adult depression mainly involves disturbances in tryptophan metabolism [7]. PUFAs are essential fatty acids that play an important role in the pathophysiology of depression. There are two main classes of PUFAs in mammals: n-3 and n-6 PUFAs. Linoleic acid (LA; C18:2 n-6) and alpha-linolenic acid (ALA; C18:3 n-3) are the most abundant dietary PUFAs. The metabolism of PUFAs depends on diet and the liver, which is considered to be the central organ for PUFA metabolism and distribution in the body. LA and ALA are converted to long-chain polyunsaturated fatty acids (LC-PUFAs), such as arachidonic acid (ARA), eicosapentaenoic acid (EPA), and docosahexaenoic acid (DHA), with a series of desaturation and elongation reactions in the liver [8,9]. EPA and DHA have been suggested as a potential treatment for depression in children and adolescents [10]. Importantly, research has also indicated that the plasma levels of EPA are decreased in patients with adolescent depression, and supplementation with EPA can improve depressive-like behavior in mice and humans [10,11,12]. Furthermore, research has demonstrated that EPA is more effective than DHA in treating depression [9,13,14], highlighting the significance of EPA in depression treatment. However, the protective role of EPA has not yet been examined in animal models of adolescent depression, and the underlying mechanism of EPA reduction in adolescent depression remains unknown.

The liver facilitates the conversion of ALA to EPA, thereby maintaining a stable environment of EPA in the brain and body [13,15], suggesting that liver dysfunction may be involved in the occurrence and development of adolescent depression. In fact, many recent studies have shown a close relationship between liver and depression [16,17,18]. For example, one-third of patients with liver cirrhosis or hepatitis exhibit symptoms of depression, and the risk of developing alcohol disorders is increased in patients with depression [19]. In addition, specific knockout of the liver soluble epoxide hydrolase (Ephx2) not only reverses chronic, mild, stress-induced depressive-like behavior but also improves synaptic function abnormalities in the prefrontal cortex [17], suggesting that the liver can directly regulate the occurrence and development of depression. However, most studies have focused on adult depression, and few studies have explored the mechanisms involving the liver in adolescents [16]. Therefore, it is essential to further investigate the effects of ELS on depression in adolescents and the potential mechanisms regulating hepatic metabolism in adolescent depression.

The main objective of this study is to investigate the effects of early-life stress on the synthesis of EPA in the liver and its subsequent impact on adolescent depression. Moreover, we aim to evaluate the potential protective role of EPA supplementation on ELS-induced depression-like behaviors in adolescent mice. Based on previous research suggesting a link among liver functionality, EPA levels, and depression, we hypothesized that ELS might disrupt liver functionality, leading to a decrease in the synthesis of EPA. This reduction could induce depression-like behaviors in adolescents. Furthermore, we proposed that supplementing EPA could potentially ameliorate these depression-like behaviors by counteracting the effects of ELS on the liver and subsequently on EPA levels. Our study aims to provide deeper insights into the mechanistic links among ELS, liver metabolism, EPA levels, and adolescent depression and explore the potential of EPA supplementation as a therapeutic strategy for adolescent depression.

## 2. Results

### 2.1. Early-Life-Stress-Induced Depression-like Behaviors Rather Than Anxiety-like Behavior in Adolescent Mice

**Figure 1:**
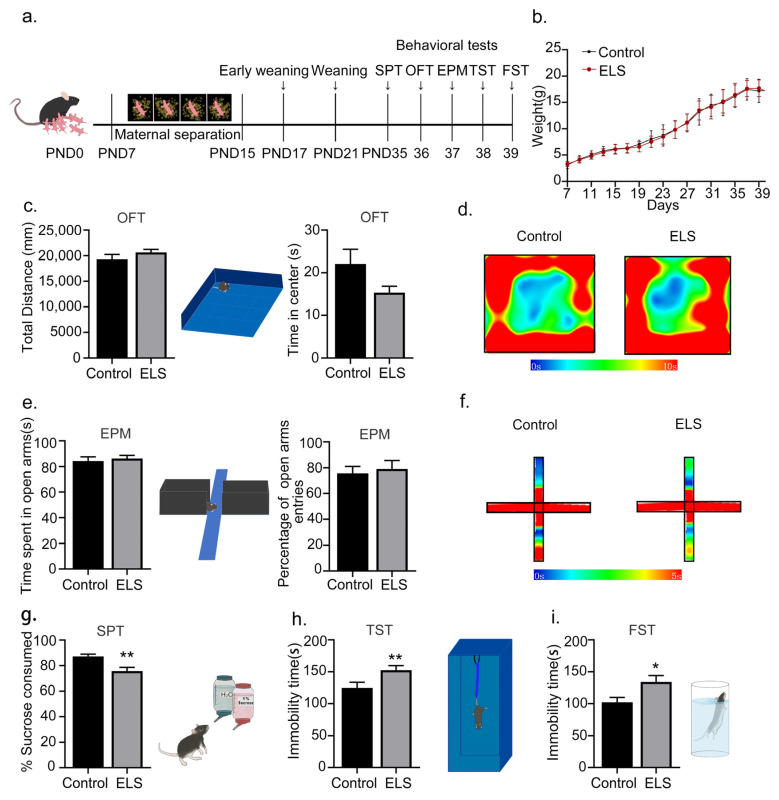
Early-life stress induced depression-like behaviors rather than anxiety-like behavior in adolescent mice: (**a**) Schematic diagram of the experimental flow. (**b**) The trend graph of the weight change of mice. (**c**) Compared with the control group, the total distance and central time of the ELS group had no significant changes. (**d**) Heat map of action trajectories in the open-field experiment. (**e**) Compared with the control group, there were no significant changes in the time spent in the open arms nor the number of entries into the open arms in the ELS group. (**f**) Heat map of action trajectories in the elevated-plus-maze test. (**g**) Compared with the control group, the percentage of sucrose consumed by the ELS group was significantly decreased. (**h**) Compared with the control group, the immobility time was significantly increased in the ELS group in the tail-suspension test. (**i**) Compared with the control group, the immobility time in the forced-swimming test was significantly increased in the ELS group. n = 11 mice per group. Data are presented as means ± SEMs. ** *p* < 0.01 and * *p* < 0.05 compared with the control group.

Previous studies have demonstrated that early-life stress can induce depressive-like symptoms, but its effects on anxiety-like behavior have not been thoroughly evaluated [20]. In this study, we extended this line of investigation to assess the impact of early-life stress on both depression-like and anxiety-like behaviors. Depressive-like behaviors were evaluated using the sucrose-preference test (SPT), the tail-suspension test (TST), and the forced-swimming test (FST). In contrast, anxiety-like behaviors were monitored using the open-field test (OFT) and the elevated-plus maze (EPM). The early-life-stress program is shown in Figure 1a. Briefly, pups were either separated from their mother from postnatal day 7 (PND7) to PND15 and weaned early on PND17 (forming the ELS group), or they were not separated and underwent standard weaning on PND21 (serving as the control group). Behavioral tests were subsequently performed from PND35 to PND39. Compared with the control group, there was no significant change in body weight in the ELS group (effect size = 0.0817, 95% CI = [−1.804, 1.513], n = 11/group, t20 = 0.1829, *p* = 0.8567; Figure 1b). In addition, there were no differences in total distance (effect size = −0.518, 95% CI = [19,292, 20,633], n = 11/group, t20 = 1.212, *p* = 0.2382; Figure 1c) nor the time spent in the center (effect size = −0.770, 95% CI = [−1.05, 14.58], n = 11/group, t20 = 1.805, *p* = 0.086; Figure 1c) in the open-field test and no significant changes in the time spent on the open arms (effect size = −0.247, 95% CI = [−1.053, 14.577], n = 11/group, t20 = 0.4775, *p* = 0.6382; Figure 1e) nor the percentage of open-arm entries (effect size = −0.166, 95% CI = [−21.031, 13.395], n = 11/group, t20 = 0.3908, *p* = 0.7001; Figure 1e). The percentage of sucrose consumed was decreased significantly in the ELS group (effect size = 1.312, 95% CI = [2.700, 14.084], n = 11/group, t20 = 3.076, *p* = 0.006; Figure 1g), and the immobility time was significantly increased in both the tail-suspension test (effect size = −1.321, 95% CI = [−56.591,−11.044], n = 11/group, t20 = 3.098, *p* = 0.006; Figure 1h) and the forced-swimming test (effect size = −1.018, 95% CI = [−61.14,−4.12], n = 11/group, t20 = 2.388, *p* = 0.027; Figure 1i), indicating that early-life stress induced depression-like behavior in adolescent mice. Taken together, these results suggest that early-life stress induces depression-like behaviors rather than anxiety-like behavior in adolescent mice.

### 2.2. The Effect of Early-Life Stress on the Transcriptional Patterns in the Liver of Adolescent Mice

**Figure 2:**
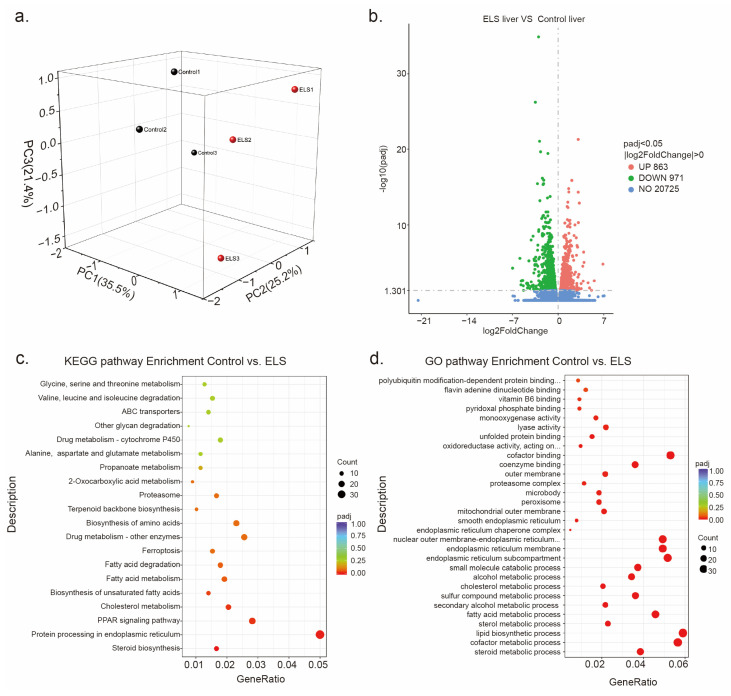
The effect of early-life stress on the transcriptional patterns in the liver of adolescent mice: (**a**) Results of principal component analysis of liver genes in ELS (cyan) and control (purple). (**b**) Volcano plot of differentially expressed genes in control and ELS group. (**c**) KEGG enrichment analysis of downregulated DEGs in control and ELS group. (**d**) GO enrichment analysis of downregulated DEGs in control and ELS group.

A substantial body of research has highlighted the significant role of the liver in depression [16,17]. Thus, we further explore the effects of early-life stress on the hepatic transcriptome changes in adolescent depression. In transcriptome sequencing, the ELS group and the control group had a total of 50.13 million and 43.96 million reads that were mapped to the reference genome sequence. Principal component analysis (PCA) was used to obtain differences between groups and intra-group sample duplications in the liver system. The significant separation between the control and ELS groups indicated that the observed changes in gene profile were associated with early-life stress (Figure 2a). An adjusted *p*-value < 0.05 was selected as the threshold value for screening the differentially expressed genes (DEGs). As shown in Figure 2b, RNA-seq revealed 863 upregulated genes and 971 downregulated genes in the liver of ELS-exposed adolescent mice. KEGG pathway enrichment analysis showed that the downregulated genes were significantly enriched in 30 pathways (Figure 2c), with the top 5 being steroid biosynthesis, protein processing in the endoplasmic reticulum, PPAR signaling pathway, cholesterol metabolism, and biosynthesis of unsaturated fatty acids. Additionally, among the 30 enriched GO pathways (Figure 2d), the top 5 were steroid metabolic process, cofactor metabolic process, lipid biosynthetic process, sterol metabolic process, and fatty acid metabolic process. These results suggest that early-life stress significantly alters the transcriptional patterns in the liver of ELS-exposed adolescent mice.

### 2.3. The Effects of Early-Life Stress on the Liver and Brain Tissue Damage in Adolescent Mice

**Figure 3:**
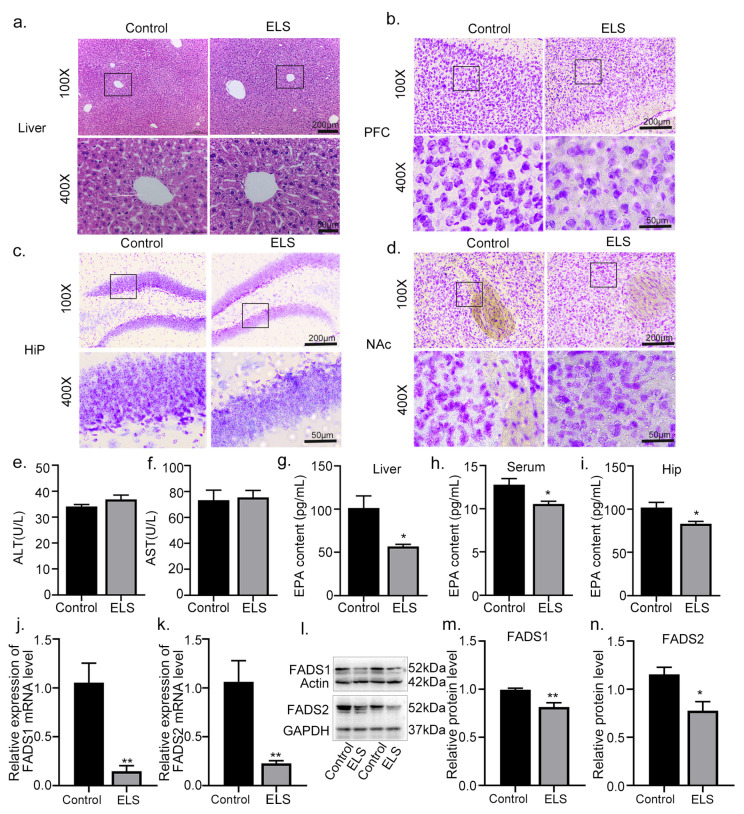
Effects of early-life stress on liver and brain tissue damage in adolescent mice: (**a**) Representative image of HE staining in the liver; (**b**–**d**) representative image of Nissl staining in the hippocampus, nucleus accumbens, and prefrontal cortex regions; (**e**,**f**) compared with the control group, there were no significant differences in the levels of ALT and AST in the ELS group (n = 5 mice per group). (**g**–**i**) Compared with the control group, the content of EPA in the liver, serum, and hippocampus was significantly decreased in the ELS group (n = 4 mice per group). (**j**,**k**). Compared with the control group, the mRNA expression of FADS1 and FADS2 in the liver was significantly decreased in the ELS group (n = 4 mice per group). (**l**–**n**). Compared with the control group, the protein level of FADS1 and FADS2 in the liver was significantly decreased in the ELS group (n = 4 mice per group). Data are presented as means ± SEMs. ** *p* < 0.01 and * *p* < 0.05 compared with the control group.

Based on our previous findings that early-life stress significantly alters hepatic transcriptomics, notably reducing the metabolism of unsaturated fatty acid biosynthesis in the liver, we proceeded to investigate the effects of early-life stress on liver–brain function and hepatic EPA levels. To assess the pathological state of liver tissue in adolescent mice, we employed hematoxylin–eosin (HE) staining to evaluate potential liver tissue damage. As depicted in Figure 2a, the HE staining results revealed no discernible injury in the liver between the ELS and control groups. Additionally, Nissl staining, which stains Nissl bodies and provides insights into cellular structures within neurons, was conducted. This staining also allows for the assessment of neuronal damage by observing Nissl bodies. As demonstrated in Figure 3b–d, no noticeable injury was observed in the medial prefrontal cortex (PFC), hippocampus (Hip), and nucleus accumbens (NAc) between the ELS group and the control group. Biochemically, the most commonly used indicator of liver injury is blood transaminase content. Thus, we detected levels of liver aspartate aminotransferase (AST) and alanine aminotransferase (ALT), and found no significant differences between the ELS and control groups in terms of ALT (effect size = −0.652, 95% CI = [−6.973, 1.525], n = 5/group, t8 = 1.478, *p* = 0.178; Figure 3e) and AST levels (effect size = −0.170, 95% CI = [−23.809, 19.469], n = 5/group, t8 = 0.2312, *p* = 0.823; Figure 3f). The current literature has reported decreased EPA content in the brains of depressed patients [7,10]. Considering this, we examined whether there was a homeostatic imbalance in the endogenous EPA conversion process in adolescent mice with depression. Our measurements of EPA content in the liver, serum, and hippocampus revealed significant reductions in the ELS group in the liver (effect size = 2.225, 95% CI = [9.900, 79.199], n = 4/group, t6 = 3.146, *p* = 0.020; Figure 3g), serum (effect size = 2.016, 95% CI = [0.313, 4.146], n = 4/group, t6 = 2.847, *p* = 0.029; Figure 3h), and hippocampus (effect size = 2.059, 95% CI = [3.01, 34.79], n = 4/group, t6 = 2.911, *p* = 0.027; Figure 3i). Fatty Acid Desaturase 1 (FADS1) and Fatty Acid Desaturase 2 (FADS2) are key rate-limiting enzymes for the conversion of ALA to EPA in the liver [8]. As indicated in Figure 3j,k, ELS significantly reduced the mRNA expression of FADS1/2 (FADS1: effect size = −3.625, 95% CI = [0.402, 1.412], n = 4/group, t6 =4.416, *p* = 0.005 (Figure 3j); FADS2: effect size = 2.704, 95% CI = [0.299, 1.370], n = 4/group, t6 = 3.789, *p* = 0.009 (Figure 3k)). Additionally, as shown in Figure 3l–n, ELS significantly decreased the protein expression of FADS1/2 (FADS1: effect size = 2.734, 95% CI = [0.065, 0.295], n = 4/group, t6 =3.828, *p* = 0.008 (Figure 3m); FADS2: effect size = 2.238, 95% CI = [0.0841, 0.671], n = 4/group, t6 = 3.152, *p* = 0.019 (Figure 3n)). These results suggest that the decreased EPA levels in the liver, serum, and hippocampus may be associated with the reduction in liver genes involved in polyunsaturated fatty acid biosynthesis, such as FADS1 and FADS2.

### 2.4. Administration of EPA Improved Depressive-like Behavior in Adolescent Mice

**Figure 4:**
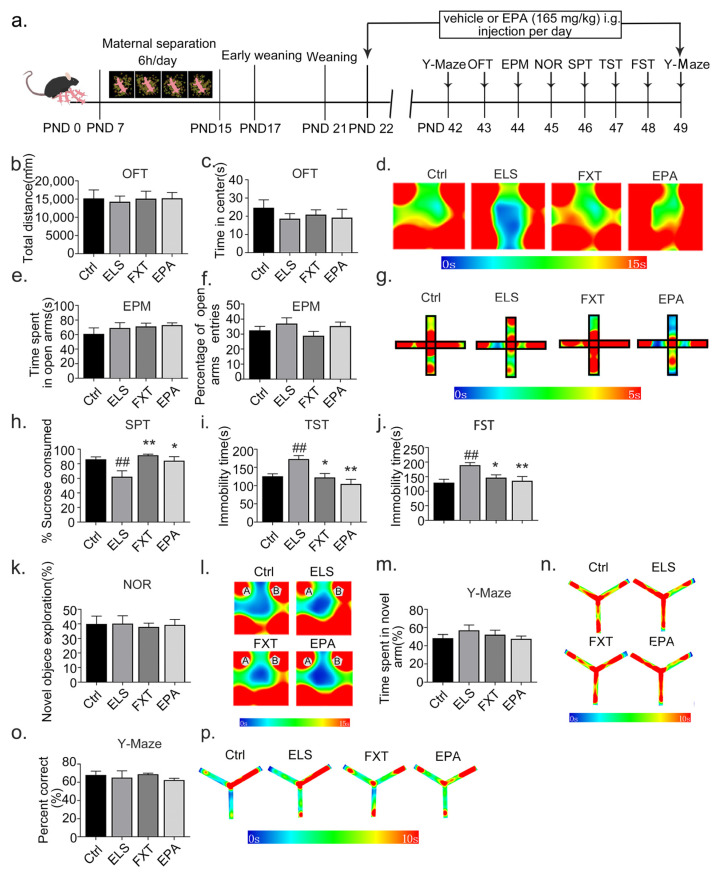
Administration of EPA improved depressive-like behavior in adolescent mice: (**a**) Schematic diagram of the experimental flow. (**b**,**c**) Compared with the ELS group, administration of EPA had no effect on the total distance and time spent in the central area in the open-field test. (**d**) Heatmap of action trajectories in the open-field experiment. (**e**,**f**) There were no significant changes in the number of entries into the open arms nor time spent in the open arms in the elevated-plus maze between groups. (**g**) Heatmap of action trajectories in the elevated-plus-maze experiment. (**h**) Compared with the ELS group, the percentage of sucrose consumed was significantly increased in the EPA group. (**i**) Compared with the ELS group, the immobility time in the tail-suspension test was significantly decreased in the EPA group. (**j**) The immobility time of the forced-swimming test was significantly reduced in EPA groups compared with the ELS group. (**k**) There was no significant change in new-object-recognition test among groups. (**l**) Action trajectories (A,B) in the new-object-recognition experiment. (**m**) There were no significant changes in short-term memory behaviors between groups. (**n**) Heat map of short-term memory behavioral trajectories in the Y-maze experiment. (**o**) There was no significant change in spontaneous alternating behavior among groups. (**p**) Heat map of the trajectory of spontaneous alternation behavior in the Y-maze experiment. n = 8 mice per group. Results are presented as means ± SEMs. ** *p* < 0.01 and * *p* <0.05 compared with ELS group; ## *p* < 0.01 compared with control group.

EPA has been found to alleviate depression-like behavior in a chronic stress-induced rat model of depression [14]. However, the impact of EPA on depression-like behaviors induced by early-life stress during adolescence remains unclear. In this study, we investigated whether EPA supplementation could ameliorate depression-like behaviors in adolescence. Specifically, EPA and DMSO (serving as vehicle controls) were administered via the intragastric route from PND22 to PND49, until the completion of the behavioral experiment. Our findings showed that EPA administration had no effects on spontaneous activity nor anxiety-like behavior (OFT total distance: effect size = 0.006, n = 8/group, F(3, 28) = 0.0578, Ctrl: 95% CI = [9662.452, 20,756.458], *p* = 0.733, FXT: 95% CI = [10,243.499, 20,025.020], *p* = 0.754, EPA:95% CI = [11,446.473, 19,028.626], *p* = 0.726 (Figure 4b); OFT time in center: effect size = 0.064, n = 8/group, F(3, 28) = 0.6360, Ctrl: 95% CI = [14.359, 34.964], *p* = 0.248, FXT: 95% CI = [14.617, 27.154], *p* = 0.644, EPA: 95% CI = [9.130, 28.247], *p* = 0.916 (Figure 4b); EPM time spent in open arms: effect size = 0.074, 95% CI = [0.065, 0.295], n = 8/group, F(3, 28) = 0.7415, Ctrl: 95% CI = [41.663, 80.324], *p* = 0.361, FXT: 95% CI = [60.314, 82.092], EPA: 95% CI = [65.511, 80.458], *p* = 0.808, *p* = 0.656 (Figure 4c); EPM percentage of open-arm entries: effect size = 0.125, 95% CI = [0.065, 0.295], n = 8/group, F(3, 28) = 1.329, Ctrl: 95% CI = [36.140, 38.177], *p* = 0.5970, FXT: 95% CI = [21.933, 35.892], *p* = 0.1750, EPA: 95% CI = [28.931, 41.720], *p* = 0.9609 (Figure 4f)). However, EPA administration resulted in a significant antidepressant effect. As illustrated in Figure 4h, EPA administration increased the percentage of sucrose consumed (effect size = 0.415, n = 8/group, F(3, 28) = 6.352, Ctrl: 95% CI = [78.450, 94.043], *p* = 0.007, FXT: 95% CI = [88.995, 96.518], *p* = 0.001, EPA: 95% CI = [71.451, 97.185], *p* = 0.014; Figure 4h) and decreased the immobility time during the tail-suspension test (effect size = 0.398, n = 8/group, F(3, 28) = 6.161, Ctrl: 95% CI = [106.070, 156.248], *p* = 0.010, FXT: 95% CI = [95.963, 154.662], *p* = 0.016, EPA: 95% CI = [73.960, 135.373], *p* = 0.000; Figure 4i) and forced-swimming test (effect size = 0.333, n = 8/group, F(3, 28) = 4.6610, Ctrl: 95% CI = [101.304, 157.717], *p* = 0.003, FXT: 95% CI = [124.053, 169.3602], *p* = 0.041, EPA: 95% CI = [104.198, 180.116], *p* = 0.009; Figure 4j).

Considering reports that acute administration of EPA can impair learning and memory function in mice [21], we further explored whether long-term administration of EPA could have similar effects. As depicted in Figure 4k–p, mice administered with EPA did not display impaired learning or memory in the novel-object recognition and Y-maze tests (novel-object exploration: effect size = 0.031, n = 8/group, F(3, 28) = 0.05318, Ctrl: 95% CI = [41.966, 79.784], *p* = 0.206, FXT: 95% CI = [65.700, 71.821], *p* = 0.469, EPA: 95% CI = [50.554, 75.791], *p* = 0.168 (Figure 4k); time spent in novel arm: effect size = 0.082, n = 8/group, F(3, 28) = 0.8366, Ctrl: 95% CI = [39.079, 57.759], *p* = 0.669, FXT: 95% CI = [40.400, 63.750], *p* = 0.572, EPA: 95% CI = [41.996, 79.784], *p* = 0.673 (Figure 4m); novel arm entries: effect size = 0.062, n = 8/group, F(3, 28) = 0.3835, Ctrl: 95% CI = [41.966, 79.784], *p* = 0.970, FXT: 95% CI = [65.700, 71.821], *p* = 0.719, EPA: 95% CI = [47.385, 69.034], *p* = 0.885 (Figure 4o)). These findings suggest that the administration of EPA ameliorates depressive-like behavior in adolescent mice.

## 3. Discussion

In the present study, we utilized a mouse model of early-life stress to explore the underlying mechanism of eicosapentaenoic acid (EPA) reduction in adolescent depression and the protective effects of EPA on adolescent depression. Our results demonstrate that ELS induced depression-like behavior rather than anxiety-like behavior in adolescent mice. Additionally, with a transcriptomic analysis of liver samples, we observed abnormal biosynthesis of unsaturated fatty acids metabolism in the liver of adolescent mice exposed to ELS and a significant reduction in the levels of EPA in the liver, serum, and hippocampus. Furthermore, ELS decreased the expression of the rate-limiting enzymes, fatty acid desaturases 1/2 (FADS1/2), involved in the biosynthesis of EPA in the liver. Additionally, EPA administration improved depression-like behavior induced by ELS, suggesting that supplementation with EPA may be beneficial to the treatment of adolescent depression.

The negative experiences encountered during early life play a significant role in the development of various emotional disorders, such as anxiety, social impairment, and learning and memory impairment [22]. Despite this, there is limited research on the emotional disorders caused by early-life stress specifically during adolescence [23,24]. In this experiment, we implemented a mother–infant separation model starting 7 days after birth (PND7), followed by single-cage separation and early weaning 17 days after birth, simulating the possible life experiences of children who are left behind in society, such as social isolation and lack of nurturing [20]. The formation and development of synapses in the brain of rodents reaches its peak on PND7, and synaptogenesis gradually ends on PND14. In addition, a recent study has shown that anesthesia-induced apoptotic neurodegeneration is the most sensitive at the peak of synaptogenesis (PND7), supporting the feasibility of using this time point for modeling in mice [25]. Adolescence is a critical period for emotional and cognitive development, and early social isolation or reduced maternal nurturing behaviors can lead to the onset of emotional disorders later in life [26,27]. We found that maternal separation with early weaning could lead to depression-like behavior rather than anxiety-like behavior in male mice during adolescence, which is in accordance with previous research [20,28,29]. Taken together, our results provide further evidence that the combination of maternal separation and early weaning represents a suitable model for investigating adolescent depression.

EPA is a polyunsaturated fatty acid that plays an important role in the organelles of neuronal cell membranes, regulating the structure and metabolism of neurons, glial cells, and endothelial cells [30]. Several lines of evidence have suggested that EPA exerts antidepressant effects by regulating neuroinflammation, normalizing astrocyte and neurotrophic in function, and regulating serotonergic neuro transmission as well as brain-derived neurotrophic factor [14,31]. Over time, evidence has suggested that the EPA content in the brain is lower in several psychiatric disorders, such as attention deficit disorder and depressive disorder, and increasing endogenous EPA content has been found to alleviate the development of these disorders [10,11,12]. Importantly, research has also indicated that the plasma levels of EPA are decreased in patients with adolescent depression [7]. Our results demonstrate that maternal separation with early weaning could also decrease EPA levels in the liver, serum, and hippocampus, and administration of EPA improved depressive-like behavior in adolescent mice, further supporting the important role of EPA in the treatment of adolescent depression. However, it is important to note that the brain’s dose of EPA is low and excessive supplementation may lead to cognitive memory impairment in humans [32]. To avoid such negative effects, we conducted Y-maze and new-object-recognition experiments to assess cognitive memory function. Our results reveal that the mice did not experience cognitive memory impairment at our chosen dose, suggesting that maintaining EPA content homeostasis is crucial to the treatment of depression-like behavior. 

EPA content in the brain is dependent on the dietary intake of ALA, which is converted in the liver and then circulates into brain cells [8,9]. Our study demonstrates that ELS caused an imbalance in the body’s EPA homeostasis but did not cause significant tissue damage to either the liver or brain tissue. Our transcriptome results further demonstrate that downregulated DEGs in the liver of ELS-exposed adolescent mice mainly participated in steroid biosynthesis, PPAR signaling pathway, fatty acid metabolism, fatty acid degradation, and unsaturated fatty acid biosynthesis, all of which are related to fatty acid regulation. In addition, we found that ELS downregulated the expression of the rate-limiting enzymes, FADS1/2, involved in the biosynthesis of EPA in the liver. A large number of studies have shown that the activity of FADS1/2 is an important rate-limiting step for ALA to synthesize EPA. And decreasing the activity of FADS1/2 desaturase could weaken the conversion of ALA to EPA [8,13,33]. Moreover, in humans carrying FADS1/2 variants, decreased levels of polyunsaturated fatty acids in various tissues have been described [34]. In addition, FADS1/2 desaturase activity plays an important role in neuropsychiatric diseases (depression, bipolar disorder, dementia), metabolism (obesity, metabolic syndrome, type 2 diabetes), and cardiovascular diseases (arterial hypertension, coronary heart disease) [33]. Thus, it is reasonable to speculate that the decreased EPA levels in the liver, serum, and hippocampus may be related to the decrease in polyunsaturated fatty acid biosynthesis in the liver. Taken together, our results provide transcriptomic evidence that ELS increases the risk of adolescent depression by reducing the synthesis of unsaturated fatty acids in the liver, especially EPA, and provide strong evidence of the potential of EPA treatment in adolescent depression associated with early-life stress.

Taken together, our results provide a deeper understanding of the mechanistic connections among liver metabolism, EPA levels, and adolescent depression. Our study, however, does have limitations. For instance, we exclusively focused on male mice; thus, our findings might not extend to female mice, as sex differences can considerably influence both the development of depression and the response to treatment. In addition, we only explored one potential mechanism—the reduction in fatty acid desaturase enzymes FADS1/2—for the observed effects of early-life stress and EPA. It is possible that other molecular and cellular mechanisms may also play a role. Further investigations are required to explore additional molecular and cellular mechanisms associated with the reduction in EPA in mice exposed to early-life stress during adolescence. Despite these limitations, our findings suggest that supplementation with EPA should be investigated as a potential treatment for adolescent depression.

## 4. Materials and Methods

### 4.1. Animals

C57BL/6 pregnant mice were obtained from Southern Medical University in Guangzhou, China. These animals were housed at Animal Research Standards Laboratory and were maintained under controlled conditions of 12-hour light/dark cycle, relative humidity between 30 and 70%, and temperature between 23 and 27 °C. The experiment was conducted in compliance with the *Guide for the Care and Use of Laboratory Animals* and was approved by the Animal Ethics Committee of Guangzhou University of Chinese Medicine, China (approval No. 20220206).

### 4.2. Early-Life-Stress Paradigm

An animal model was utilized to investigate the effects of early-life stress on adolescent depression. Early-life stress consisted of a combination of maternal separation and an early weaning on PND17 [20]. After birth, mice were randomly allocated to two groups: control and early-life-stress group. Pups of the early-life-stress group were separated from their mothers between postnatal days 7 and 15 (PND7–15) and isolated in a new cage with appropriate bedding, water, and food for 6 h per day, and these pups were weaned on PND17. On PND21, 11 male pups were randomly assigned to the ELS group. The pups of the control group were not manipulated until regular weaning on P21, and on PND 21, 11 male pups were randomly assigned to the control group. Male mice were used for subsequent behavioral tests, mainly to avoid the potential confounding effects of cyclical hormonal fluctuations inherent in females, including estrogen [35]. These fluctuations may affect the behavioral response.

### 4.3. Drug Administration

Fluoxetine was given at a clinically equivalent dose of 5.0 mg/kg in mice based on previous studies [36]. The EPA (HY-B0660; MedChemExpress, Monmouth Junction, NJ, USA) concentrations used were based on the International Society for Nutritional Psychiatry Practice Guidelines for the Study of Omega-3 Fatty Acids in the treatment of major depressive disorder, with the dosage calculated based on an adult body weight of 75 kg. EPA was dissolved in dimethyl sulfoxide (DMSO) and administered at a final concentration of 0.1% DMSO. EPA was administered intragastrically at the dose of 165 mg/kg based on previous studies [10,12]. The mice were randomly divided into four groups: control, ELS, ELS + Fluoxetine, and ELS + EPA. The control mice were reared under normal conditions, while mice in the ELS groups were subjected to maternal separation with early weaning. Fluoxetine, EPA, and DMSO (as a vehicle control) were administered via intragastric route from PND22 to PND49, until the conclusion of the behavioral experiment. All drugs were freshly prepared.

### 4.4. Anxiety-like Behavior Test

Anxiety-like behavior was assessed with the open-field test (OFT) and the elevated-plus maze (EPM) [20,37,38]. The procedure of the OFT involved placing each mouse at the center of an open field in a dimly lit room and recording their trajectory for 10 min using a video camera. Subsequently, the EPM was conducted, in which the animals were placed at the center of a cross elevation apparatus facing the open-arm direction, and their trajectory was recorded for 6 min in a dimly lit room. The criteria for entering the open arm and the closed arm were based on the mice entering with 2/3 of the body volume. All results were analyzed using SuperMaze software (version 2.0; Shanghai Xinruan Information Tech Co., Shanghai, China). The experimenters were blinded to the treatments during the scoring process.

### 4.5. Depression-like Behavior Test

Depression-like behavior was assessed with the sucrose-preference test (SPT), the tail-suspension test (TST), and the forced-swimming test (FST) according to previous studies [20,37,38]. The tail-suspension experiment was performed by hanging the mice with tape about 1 cm from the tip of the tail and 25 cm from the ground and recording the resting time and latency period for 6 min. The forced-swimming experiment involved placing the mice in a cylindrical container containing 25 °C water at a depth of 25 cm, and recording the duration of the immobile state for 4 min after 6 min. All results were analyzed using SuperMaze software (version 2.0; Shanghai Xinruan Information Tech Co., Shanghai, China). The experimenters were blinded to the treatments during the scoring process.

### 4.6. Learning and Memory Behavior Test

In order to evaluate the effects of early-life stress and EPA administration on learning and memory, the Y-maze experiment and the new-object-recognition experiment were used according to our previous study [39]. We evaluated the spatial working memory of mice using a spontaneous alternation experiment. The Y-maze consists of three arms at 120° angles from each other. The mouse was gently lifted by its tail, facing the wall of the arm, and randomly placed at the end of one arm, allowing it to freely explore the three open arms for 5 min. We recorded the order in which the mouse sequentially entered the three arms. Entering the three arms in a clockwise or counterclockwise order was defined as correct times, whereas alternating order of entering the three arms was defined as incorrect times. The spontaneous alternation rate was calculated as the correct times divided by the maximum possible alternation times (total times minus 2) times 100%. Seven days after the conclusion of the spontaneous alternation experiment, we further evaluated the mice’s short-term spatial memory using a Y-maze test. The experiment included a training period and a testing period. During the training period, one arm of the maze was closed off as the novel arm, with the other two arms were left open. One of these open arms was designated as the starting arm. Mice were placed into the starting arm and allowed to freely navigate for 5 min. After 15 min, all arms of the maze were opened, and the mice were allowed to freely explore the three arms for another 5 min. We recorded the time spent in each arm and calculated the percentage of time spent in the novel arm to evaluate short-term spatial memory.

The novel-object recognition experiment was conducted in an open-field box over a period of three days, including three stages: habituation, training, and testing. During the habituation phase, the mouse was gently lifted by the tail and placed in the center of the open-field box to freely explore for 10 min. On the second day, two different objects (A and B) were placed in the open-field box, and the mouse was allowed to freely explore for 5 min. In the testing phase, object B was replaced with a new object C, and the time the mouse spent exploring objects A and C was recorded. The index for evaluating novel-object recognition memory is the exploration preference index (the time spent by the mouse exploring the new object divided by the total exploration time of the old and new objects). The experimenters were blinded to the treatments during the scoring process.

### 4.7. RNA-Sequencing Analysis

Twenty-four hours after the behavioral tests, the liver tissues were collected from mice [39]. The liver tissues (n = 3 per group) were sent to Beijing Nuohe Zhiyuan biological information technology Co., Ltd., to prepare libraries for sequencing. We used clusterProfiler (3.4.4) software for Gene Ontology (GO) enrichment analysis and Kyoto Encyclopedia of Gene and Genomes (KEGG) analysis of differentially expressed genes. 

### 4.8. AST and AST Assay

Aspartate amino transaminase and alanine amino transaminase kits were procured from Nanjing Jiancheng Institute of Biological Engineering. Blood samples were collected from the orbital region of the mice using EDTA-coated tubes and allowed to sit at room temperature for 60 min. The samples were then centrifuged at 12,000 revolutions per minute for 10 min, and the supernatant was collected for analysis. The levels of AST and ALT in the serum of each group of mice were determined according to the instructions provided in the kit.

### 4.9. Hematoxylin–Eosin Staining

Hematoxylin–eosin staining was performed as previously described [39,40]. The hematoxylineosin staining process followed these steps: Paraffin sections were subjected to sequential treatment with xylene, graded ethanol, and distilled water. They were then stained with hematoxylin for 10 min and washed with tap water for another 10 min. Subsequently, the sections were briefly differentiated with 0.1% hydrochloric alcohol, rinsed with water, and eosin-stained for 10 min. After staining, the sections underwent dehydration, were cleared with xylene, and were slightly air-dried. Finally, the sections were sealed with neutral balsam.

### 4.10. Nissl Staining

Nissl staining was performed as previously described [38,40]. The procedure for Nissl staining was executed as follows: Brain sections underwent a process of deparaffinization and rehydration, which involved two stages of xylene treatment for 10 min each, followed by sequential alcohol dehydration (100% for 5 min, 90% for 5 min, and 70% for 5 min). The sections were then washed with distilled water for 2 min. The staining was achieved using Nissl staining solution (C0117; Beyotime, Shanghai, China) for a period of 10 min. Following staining, the sections were washed twice with distilled water for a few seconds each time. Dehydration was then resumed in 70% and 95% ethanol. The sections were rendered transparent with xylene and subsequently inspected under an optical microscope.

### 4.11. EPA Assay

The measurement of EPA levels was performed using an enzyme-linked immunosorbent assay kit (MM-45902M2; Jiangsu Meimian industrial Co., Ltd., Yancheng, China) according to the manufacturer’s instructions. To obtain the necessary samples, mouse orbital blood was collected in sterile EP tubes and allowed to clot at room temperature for 20 min. The samples were then centrifuged at 2–8 °C for 20 min at a rotation speed of 6072 g using a high-speed refrigerated centrifuge (3-188; Hunan kecheng Instrument Co., Changsha, China), and the resulting supernatant was collected and set aside. Similarly, mouse hippocampal and liver tissue samples were obtained, sectioned, and stored at −80 °C for future analysis. The tissue samples were homogenized in phosphate-buffered saline (PBS) and centrifuged for 20 min at 6072 g (3-188; Hunan kecheng Instrument Co.), and the supernatant was collected. The samples were then analyzed according to the instructions provided in the ELISA kit, and the levels of EPA were determined for each group of mice.

### 4.12. Western Blot (WB)

Western blots were performed as previously described [39]. The primary antibodies were FADS1 (1:1000; ab126706), FADS2 (1:1000; Proteintech 28034-1-AP), GAPDH (1:5000; Affinity AF7021), actin (1:5000; Proteintech 60008-1-Ig). Then, the corresponding secondary antibodies were incubated at room temperature for 1 h. The bound antibody was expressed using the ECL method and detected with the BIO-RID imaging system (BIO-RAD, Hercules, CA, USA). The grayscale values of all bands were analyzed with Image Lab (Image J 1.53C, Millipore, Burlington, MA, USA) software.

### 4.13. Quantitative Real-Time Polymerase Chain Reaction (q-PCR)

In order to perform quantitative analysis, total RNA was extracted from liver tissue samples using Trizol reagent according to our previous study [39]. The primers used for the amplification were designed based on the relevant literature and were confirmed with the PrimerBank or GenBank gene library to avoid the formation of stable dimers or hairpin structures. Analysis was performed using the ΔΔC(t) method. The specific primers used for the FADS1 gene were upstream 5′-TGGTGGAACCACTTGCACTT-3′ and downstream 5′-TTCTG TTCCCGAGCTCCAC-3′, with an amplification length of 128 bp. The specific primers used for the FADS2 gene were upstream 5′-TGACCGCAAGG TTTACAACAT-3′ and downstream 5′-AGGCATCCGTTGCATAT-3′. The specific primers used for the GAPDH gene were upstream 5′-ACGGCAAATTCAACGGCACAG-3′ and downstream 5′-ACACCAGTAGACTCCACGACATAC-3′. The reaction conditions included a pre-denaturation step at 95 °C for 30 s, followed by a denaturation step at 95 °C for 5 s, an annealing step at 60 °C for 34 s, and 40 cycles of the following steps: 95 °C for 15 s, 60 °C for 1 min, and 95 °C for 15 s. The reaction was then held at 4 °C.

### 4.14. Statistical Analysis

Statistical analysis was conducted using SPSS 20.0 software. Multiple groups were compared using one-way ANOVA, followed by Fisher’s Least Significant Difference (LSD) post hoc test for pairwise comparisons. Two groups were performed using Student’s *t*-tests. The significance level was set at *p* < 0.05. All data were expressed as means ± standard errors. 

## 5. Conclusions

Our findings offer transcriptomic evidence suggesting that early-life stress increases the risk of adolescent depression by reducing the synthesis of unsaturated fatty acids, particularly EPA, in the liver. These results indicate that supplementation with EPA should be investigated as a potential treatment for adolescent depression.

## Data Availability

The datasets used and analyzed during the current study are available from the corresponding authors upon request.

## References

[1] LeMoult J., Humphreys K.L., Tracy A., Hoffmeister J.A., Ip E., Gotlib I.H. (2020). Meta-analysis: Exposure to Early Life Stress and Risk for Depression in Childhood and Adolescence. J. Am. Acad. Child. Adolesc. Psychiatry.

[2] Fang X., Fry D.A., Ji K., Finkelhor D., Chen J., Lannen P., Dunne M.P. (2015). The burden of child maltreatment in China: A systematic review. Bull. World Health Organ..

[3] Hung C.I., Yu N.W., Liu C.Y., Wu K.Y., Yang C.H. (2015). The impact of the duration of an untreated episode on improvement of depression and somatic symptoms. Neuropsychiatr. Dis. Treat..

[4] Clark M.S., Jansen K.L., Cloy J.A. (2012). Treatment of childhood and adolescent depression. Am. Fam. Physician.

[5] Solmi M., Fornaro M., Ostinelli E.G., Zangani C., Croatto G., Monaco F., Krinitski D., Fusar-Poli P., Correll C.U. (2020). Safety of 80 antidepressants, antipsychotics, anti-attention-deficit/hyperactivity medications and mood stabilizers in children and adolescents with psychiatric disorders: A large scale systematic meta-review of 78 adverse effects. World Psychiatry.

[6] Masi G. (2022). Controversies in the Pharmacotherapy of Adolescent Depression. Curr. Pharm. Des..

[7] Zhou X., Liu L., Lan X., Cohen D., Zhang Y., Ravindran A.V., Yuan S., Zheng P., Coghill D., Yang L. (2019). Polyunsaturated fatty acids metabolism, purine metabolism and inosine as potential independent diagnostic biomarkers for major depressive disorder in children and adolescents. Mol. Psychiatry.

[8] Kalkman H.O., Hersberger M., Walitza S., Berger G.E. (2021). Disentangling the Molecular Mechanisms of the Antidepressant Activity of Omega-3 Polyunsaturated Fatty Acid: A Comprehensive Review of the Literature. Int. J. Mol. Sci..

[9] Yalagala P.C.R., Sugasini D., Dasarathi S., Pahan K., Subbaiah P.V. (2019). Dietary lysophosphatidylcholine-EPA enriches both EPA and DHA in the brain: Potential treatment for depression. J. Lipid. Res..

[10] Chang J.P., Su K.P. (2020). Nutritional Neuroscience as Mainstream of Psychiatry: The Evidence- Based Treatment Guidelines for Using Omega-3 Fatty Acids as a New Treatment for Psychiatric Disorders in Children and Adolescents. Clin. Psychopharmacol. Neurosci..

[11] Choi J.E., Park Y. (2017). EPA and DHA, but not ALA, have antidepressant effects with 17beta-estradiol injection via regulation of a neurobiological system in ovariectomized rats. J. Nutr. Biochem..

[12] Guu T.W., Mischoulon D., Sarris J., Hibbeln J., McNamara R.K., Hamazaki K., Freeman M.P., Maes M., Matsuoka Y.J., Belmaker R.H. (2019). International Society for Nutritional Psychiatry Research Practice Guidelines for Omega-3 Fatty Acids in the Treatment of Major Depressive Disorder. Psychother. Psychosom..

[13] Bazinet R.P., Metherel A.H., Chen C.T., Shaikh S.R., Nadjar A., Joffre C., Laye S. (2020). Brain eicosapentaenoic acid metabolism as a lead for novel therapeutics in major depression. Brain Behav. Immun..

[14] Peng Z., Zhang C., Yan L., Zhang Y., Yang Z., Wang J., Song C. (2020). EPA is More Effective than DHA to Improve Depression-Like Behavior, Glia Cell Dysfunction and Hippcampal Apoptosis Signaling in a Chronic Stress-Induced Rat Model of Depression. Int. J. Mol. Sci..

[15] Valentini K.J., Pickens C.A., Wiesinger J.A., Fenton J.I. (2018). The effect of fish oil supplementation on brain DHA and EPA content and fatty acid profile in mice. Int. J. Food Sci. Nutr..

[16] Wang B., Lu S., Zhang C., Zhu L., Li Y., Bai M., Xu E. (2020). Quantitative proteomic analysis of the liver reveals antidepressant potential protein targets of Sinisan in a mouse CUMS model of depression. Biomed. Pharmacother..

[17] Qin X.H., Wu Z., Dong J.H., Zeng Y.N., Xiong W.C., Liu C., Wang M.Y., Zhu M.Z., Chen W.J., Zhang Y. (2019). Liver Soluble Epoxide Hydrolase Regulates Behavioral and Cellular Effects of Chronic Stress. Cell Rep..

[18] Kahl K.G., Krüger T., Eckermann G., Wedemeyer H. (2019). Major depression and liver disease: The role of microbiome and inflammation. Fortschr. Neurol. Psychiatr..

[19] Hernaez R., Kramer J.R., Khan A., Phillips J., McCallister K., Chaffin K., Hernandez A.P., Fullington H., Ortiz C., Blackwell J.-M. (2022). Depression and Anxiety Are Common Among Patients With Cirrhosis. Clin. Gastroenterol. Hepatol..

[20] Tchenio A., Lecca S., Valentinova K., Mameli M. (2017). Limiting habenular hyperactivity ameliorates maternal separation-driven depressive-like symptoms. Nat. Commun..

[21] Chang J.P.-C., Su K.-P., Mondelli V., Satyanarayanan S.K., Yang H.-T., Chiang Y.-J., Chen H.-T., Pariante C.M. (2019). High-dose eicosapentaenoic acid (EPA) improves attention and vigilance in children and adolescents with attention deficit hyperactivity disorder (ADHD) and low endogenous EPA levels. Transl. Psychiatry.

[22] Featherstone R.E., Gifford R.L., Crown L.M., Amirfathi F., Alaniz J.P., Yi J., Tran A., Adomian D., Schwenk A., Melnychenko O. (2022). Early life social instability stress causes lasting cognitive decrement and elevated hippocampal stress-related gene expression. Exp. Neurol..

[23] Qin X., Liu X.-X., Wang Y., Wang D., Song Y., Zou J.-X., Pan H.-Q., Zhai X.-Z., Zhang Y.-M., Zhang Y.-B. (2021). Early life stress induces anxiety-like behavior during adulthood through dysregulation of neuronal plasticity in the basolateral amygdala. Life Sci..

[24] Catale C., Bisicchia E., Carola V., Viscomi M.T. (2021). Early life stress exposure worsens adult remote microglia activation, neuronal death, and functional recovery after focal brain injury. Brain Behav. Immun..

[25] Yon J.H., Daniel-Johnson J., Carter L.B., Jevtovic-Todorovic V. (2005). Anesthesia induces neuronal cell death in the developing rat brain via the intrinsic and extrinsic apoptotic pathways. Neuroscience.

[26] Lee J.-S., Kang J.-Y., Son C.-G. (2020). A Comparison of Isolation Stress and Unpredictable Chronic Mild Stress for the Establishment of Mouse Models of Depressive Disorder. Front. Behav. Neurosci..

[27] Kwong A.S.F., López-López J.A., Hammerton G., Manley D., Timpson N.J., Leckie G., Pearson R.M. (2019). Genetic and Environmental Risk Factors Associated With Trajectories of Depression Symptoms From Adolescence to Young Adulthood. JAMA Netw. Open.

[28] Alteba S., Portugalov A., Hillard C.J., Akirav I. (2021). Inhibition of Fatty Acid Amide Hydrolase (FAAH) During Adolescence and Exposure to Early Life Stress may Exacerbate Depression-like Behaviors in Male and Female Rats. Neuroscience.

[29] Chen Y., Zheng Y., Yan J., Zhu C., Zeng X., Zheng S., Li W., Yao L., Xia Y., Su W.-W. (2021). Early Life Stress Induces Different Behaviors in Adolescence and Adulthood May Related With Abnormal Medial Prefrontal Cortex Excitation/Inhibition Balance. Front. Neurosci..

[30] Kapoor B., Kapoor D., Gautam S., Singh R., Bhardwaj S. (2021). Dietary Polyunsaturated Fatty Acids (PUFAs): Uses and Potential Health Benefits. Curr. Nutr. Rep3..

[31] Wang C.-C., Du L., Shi H.-H., Ding L., Yanagita T., Xue C.-H., Wang Y.-M., Zhang T.-T. (2021). Dietary EPA-Enriched Phospholipids Alleviate Chronic Stress and LPS-Induced Depression- and Anxiety-Like Behavior by Regulating Immunity and Neuroinflammation. Mol. Nutr. Food Res..

[32] Liu J.-H., Wang Q., You Q.-L., Li Z.-L., Hu N.-Y., Wang Y., Jin Z.-L., Li S.-J., Li X.-W., Yang J.-M. (2020). Acute EPA-induced learning and memory impairment in mice is prevented by DHA. Nat. Commun..

[33] Zak A., Slaby A., Tvrzicka E., Jachymova M., Macasek J., Vecka M., Zeman M., Stankova B. (2016). Desaturases of fatty acids (FADS) and their physiological and clinical implication. Cas. Lek. Cesk..

[34] Lattka E., Illig T., Koletzko B., Heinrich J. (2010). Genetic variants of the FADS1 FADS2 gene cluster as related to essential fatty acid metabolism. Curr. Opin. Lipidol..

[35] Palanza P., Parmigiani S. (2017). How does sex matter? Behavior, stress and animal models of neurobehavioral disorders. Neurosci. Biobehav. Rev..

[36] Deng D., Cui Y., Gan S., Xie Z., Cui S., Cao K., Wang S., Shi G., Yang L., Bai S. (2022). Sinisan alleviates depression-like behaviors by regulating mitochondrial function and synaptic plasticity in maternal separation rats. Phytomedicine.

[37] Zhao J., Ye L., Liu Z., Cui Y., Deng D., Bai S., Yang L., Shi Y., Liu Z., Zhang R. (2022). Protective Effects of Resveratrol on Adolescent Social Isolation-Induced Anxiety-Like Behaviors via Modulating Nucleus Accumbens Spine Plasticity and Mitochondrial Function in Female Rats. Nutrients.

[38] Cui S., Lin H., Cui Y., Wen W., Cui X., Shen C., Mo H., Yang L., Bai S., Shi Y. (2021). Depression promotes lung carcinoma progression by regulating the tumor microenvironment in tumor-bearing models of C57BL/6J mice. Neurosci. Lett..

[39] Zhao J., Ying L., Liu Y., Liu N., Tu G., Zhu M., Wu Y., Xiao B., Ye L., Li J. (2019). Different roles of Rac1 in the acquisition and extinction of methamphetamine-associated contextual memory in the nucleus accumbens. Theranostics.

[40] Shen C., Cao K., Cui S., Cui Y., Mo H., Wen W., Dong Z., Lin H., Bai S., Yang L. (2020). SiNiSan ameliorates depression-like behavior in rats by enhancing synaptic plasticity via the CaSR-PKC-ERK signaling pathway. Biomed. Pharmacother..

